# Quantitative Visualization of Buried Defects in GFRP via Microwave Reflectometry

**DOI:** 10.3390/s23146629

**Published:** 2023-07-24

**Authors:** Ruonan Wang, Yang Fang, Qianxiang Gao, Yong Li, Xihan Yang, Zhenmao Chen

**Affiliations:** State Key Laboratory for Strength and Vibration of Mechanical Structures, Shaanxi Engineering Research Centre of NDT and Structure Integrity Evaluation, School of Aerospace Engineering, Xi’an Jiaotong University, Xi’an 710049, China; w2502209459@stu.xjtu.edu.cn (R.W.); gaoqianxiang@stu.xjtu.edu.cn (Q.G.); yxihan@stu.xjtu.edu.cn (X.Y.); chenzm@mail.xjtu.edu.cn (Z.C.)

**Keywords:** electromagnetic nondestructive testing, microwave reflectometry, glass fiber-reinforced polymer, buried defects, quantitative evaluation

## Abstract

Glass fiber-reinforced polymer (GFRP) is widely used in engineering fields involving aerospace, energy, transportation, etc. If internal buried defects occur due to hostile environments during fabrication and practical service, the structural integrity and safety of GFRP structures would be severely undermined. Therefore, it is indispensable to carry out effective quantitative nondestructive testing (NDT) of internal defects buried within GFRP structures. Along with the development of composite materials, microwave NDT is promising in non-intrusive inspection of defects in GFRPs. In this paper, quantitative screening of the subsurface impact damage and air void in a unidirectional GFRP via microwave reflectometry was intensively investigated. The influence of the microwave polarization direction with respect to the GFRP fiber direction on the reflection coefficient was investigated by using the equivalent relative permittivity calculated with theoretical analysis. Following this, a microwave NDT system was built up for further investigation regarding the imaging and quantitative evaluation of buried defects in GFRPs. A direct-wave suppression method based on singular-value decomposition was proposed to obtain high-quality defect images. The defect in-plane area was subsequently assessed via a proposed defect-edge identification method. The simulation and experimental results revealed that (1) the testing sensitivity to buried defects was the highest when the electric-field polarization direction is parallel to the GFRP fiber direction; and (2) the averaged evaluation accuracy regarding the in-plane area of the buried defect reached approximately 90% by applying the microwave reflectometry together with the proposed processing methods.

## 1. Introduction

Because of the excellent flexural strength, high stiffness-to-weight ratio, and outstanding corrosion resistance and thermal insulation [1,2,3], glass fiber-reinforced polymer (GFRP) is considered one of the preferable structural materials for fuselage skins, concrete reinforced beams, transportation pipelines, etc. [4,5,6]. Hitherto, it has been extensively utilized in structures of aerospace, automotive, marine, transportation, infrastructure, and other engineering fields [7,8]. However, it is inevitable that the GFRP structure is vulnerable to structural defects that undermine the stiffness as well as strength of structures, and pose a severe threat to the integrity of GFRP. Therefore, it is imperative to conduct nondestructive testing (NDT) of defects in GFRP structures in an effort to ensure the safety of engineering structures.

It is noteworthy that among typical defects of GFRP structures, the buried defect is deemed as one of the most critical flaws. This is because the buried defect normally conceals itself either within the structure body (manifesting itself as the void normally occurring during fabrication) or on the back surface of the structure (manifesting itself as the localized back surface material loss normally occurring during practical service due to the lateral impact [9]). The characteristics of the buried defect and GFRP make the traditional nondestructive testing techniques such as visual testing and eddy current testing, etc. formidable for quantitative visualization of defects. Although the testing methods including ultrasonic testing [10,11], infrared testing [12], and radiographic testing [13] are currently adopted for GFRP inspection, they could have technical drawbacks, particularly regarding the evaluation of the buried defect. Though air-coupled ultrasonic testing barely needs coupling [11], in the air, high-frequency signals decay rapidly, and thus the testing sensitivity to the buried defect could be low. The disadvantage of infrared testing lies in the fact that the accuracy in the detection of the buried defect could be limited due to the low thermal conductivity of GFRP. The major limitations of radiographic testing involve high costs, slow testing, and radiation hazards.

Compared with the aforementioned testing techniques, microwave testing has attracted much attention in the NDT field, especially regarding quantitative inspection of dielectric structures. The advantages of microwave testing include non-contact and remote testing, low attenuation in non-conductive materials, and one-side inspection of defects [14]. Besides, high-frequency electromagnetic waves with wide frequency bands are used in defect detection, making the testing capable of efficient imaging of hidden flaws with high longitudinal and transverse resolutions. As a result, microwave testing is found to have good detection accuracy for volumetric defects such as wall-thinning defects, and interlaminar flaws such as debonding and delamination in single-layer/multi-layer dielectric structures. Li et al. combined near-field and far-field microwave scanning imaging to obtain the full-size defect image of the large stiffened composite structure, greatly improving the detection efficiency [15]. Mei et al. developed an automatic detection system for full-size composite insulators and air-gap defects, carbonization defects, and conductive defects were located [16]. Wang et al. detected a phase change paraffin inclusion in honeycomb sandwich panels in the near field and accurately characterized the boundary of the phase change inclusion [17]. In addition, microwave testing can also be applicable to the detection of surface cracks in metallic structures. Yu et al. investigated the imaging and recognition of the boundary of the corrosion cracks in aluminum alloy structure via microwave testing [18]. Studies regarding microwave-testing-based GFRP evaluation have also been conducted. Rahman et al. compared the detection performance of K-band circular, K-band, and Ka-band rectangular waveguide for external and internal defects in GFRP pipe [19]. Shrifan et al. presented a novel technique based on k-means unsupervised machine learning with K-band rectangular waveguide to identify defects in GFRP [20]. In the study by Wang et al., a microwave equiphase frequency truncation method was proposed to detect and evaluate the thickness of kissing defects in GFRP laminates [21]. Sutthaweekul et al. combined principal component analysis and synthetic aperture radar to detect and characterize flat-bottom holes in coated GFRP pipes [22]. Shrifan et al. used maximal overlap discrete wavelet packet transform and the Bi-directional long short-term memory network approach to distinguish disbands in GFRP with rectangular waveguide operating from 18 to 26.5 GHz [23].

From the publications, it can be noticed that as one of the microwave antennae, the rectangular waveguide is often used for the detection of defects in GFRP. However, to the authors’ knowledge, few studies involve the influence of the anisotropic characteristics of GFRP on the testing results. For a rectangular waveguide with TE_10_ mode as the fundamental mode of propagation, the direction of the electric field in the radiated microwave, i.e., polarization direction is parallel to the short edge of the waveguide aperture. When a unidirectional GFRP is inspected by the rectangular waveguide, the fiber direction of the GFRP has a certain angle concerning the wave-polarization direction. According to relevant research [24], when the included angle is different, the relative permittivity of GFRP in the detection system changes accordingly. This implies that (1) the anisotropic characteristics of GFRP influence the testing results; and (2) with the angle known, the so-called equivalent permittivity of GFRP, which can be computed with the corresponding formula mentioned in reference [25], could be applied for investigation regarding the influence of the wave-polarization direction with respect to the GFRP fiber direction on the reflection coefficient.

In this paper, the detection, imaging, and quantitative assessment of buried defects in a unidirectional GFRP slab via microwave reflectometry, i.e., one of the measurement methods in microwave testing, were intensively investigated. Two typical angles between the microwave polarization direction and the GFRP fiber direction, i.e., parallel and orthogonal, were taken into account. Numerical simulation models for microwave testing of buried defects were established in an attempt to scrutinize the testing signal response to the defect with the derived equivalent dielectric constant from the theoretical study. An experimental system of Ka-band microwave testing and screening of GFRP structures was built up. The testing signal and defect images for two angle scenarios were further analyzed. A direct-wave suppression method based on single-value sequence optimization was proposed for the enhancement of the defect image quality. Based on the defect image, the subsequent process involving image interpolation, feature enhancement, and defect-edge recognition is realized in an effort to evaluate the in-plane area of the buried defect.

## 2. Theoretical Analysis and Simulations

### 2.1. Theoretical Analysis of Microwave Response to a Dielectric Material

**Figure 1:**
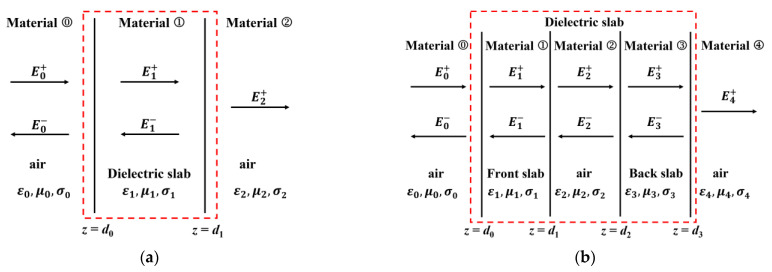
Electromagnetic wave propagation for microwave detection of buried defects: (**a**) electromagnetic wave propagation for the scenario with the back surface material loss; (**b**) electromagnetic wave propagation for the case with the internal hole.

Microwave reflectometry is based on high-frequency electromagnetic waves which are emitted from microwave probes/antennas, and usually scan the specimens under inspection at the normal incident angle. During wave propagation, the incident wave is reflected at each heterogeneous interface. Since the reflected wave is closely associated with the material properties and sizing parameters, by analyzing the resulting testing signal the parameters of defects in the specimen can be extracted [26]. Since GFRP is non-conductive, it can be taken as one of the dielectric materials. Theoretical analysis is conducted to investigate the microwave response to a dielectric slab subject to the buried defect with a length and width appreciably larger than the size of the microwave antenna aperture. It is assumed that the microwave generated by an antenna is the transverse electric and magnetic field (TEM) plane wave that propagates along the *z* direction in the Fraunhofer region. The wave-propagation model is exhibited in Figure 1. Because the electric-field direction, magnetic-field direction, and wave-propagation direction are orthogonal to each other, based on Maxwell’s equations, the expression regarding the *x-*component of the electric field of TEM wave propagating within a lossless non-conducting medium can be formulated as Equation (1) [26]:

(1) $$E_{xi}\left(z\right)=E_{xi}^{+}e^{-jkz}+E_{xi}^{-}e^{jkz},k_{i}=\omega \sqrt{\mu_{i}\varepsilon_{i}}$$

where, the subscript *i* represents different dielectric material domains, and $E_{x}^{+}$ and $E_{x}^{-}$ denote the electric-field intensities regarding the incident and reflected microwaves at the boundary of *z* = 0, respectively. *k* and *ω* stand for the wave number and angular frequency, respectively. *μ* is the permeability of the medium, which is essentially the product of the vacuum permeability and medium relative permeability. *ε* denotes the medium permittivity which is the product of the vacuum permittivity and relative permittivity of the medium. It is noted that for a plane TEM wave the *y* and *z* components of the electric field vanish. In regard to the magnetic field of the TEM wave, its *y* component can be written as Equation (2) [26]:

(2) $$H_{yi}^{+}\left(z\right)=\frac{1}{\eta_{i}}E_{xi}^{+}\left(z\right),\eta_{i}=\sqrt{\frac{\mu_{i}}{\varepsilon_{i}}}$$

where, *η* is the intrinsic impedance of the medium. Note that the *x* and *z* components of the magnetic field vanish.

Based on the model, the buried defects involving the back surface material loss and internal hole (simulating the void) can be modeled by either varying the thickness of the slab, i.e., *d* or introducing a planar air layer (with the thickness of *d*_1_ − *d*_2_) to the slab. When the slab is tested by the incident microwave, for the scenario of the back surface material loss in Figure 1a, based on Equations (1) and (2) in conjunction with the continuity of the electromagnetic field at *z* = 0 and *z* = *d*_1_, the intensity of the electric field of the reflected wave $E_{0}^{-}$ can be formulated as Equation (3):

(3) $$\left\{\begin{array}{l} E_{0}^{-}=cE_{0}^{+}\\ c=-\frac{j\left(\eta_{2}{}^{2}-\eta_{1}{}^{2}\right)\tan(k_{1}d_{1})}{2\eta_{1}\eta_{2}+j\left(\eta_{1}{}^{2}+\eta_{2}{}^{2}\right)\tan(k_{1}d_{1})} \end{array}\right.$$

where, *c* denotes the reflection coefficient of the slab subject to the back surface material loss. *η*_1_ and *η*_2_ are the natural impedances of the air and flawless slab, respectively. *k*_1_ is the wave number in the slab domain. It can be noticed from Equation (3) that the reflection coefficient *c* is highly associated with the variation in the slab thickness. By analyzing the reflection coefficient which is usually measured in microwave testing, the back surface material loss could be detected and evaluated.

For the scenario regarding the internal hole in Figure 1b, the relation of the reflected wave with the incident wave can be expressed in the matrix notation as Equation (4):

(4) $$\left[\begin{array}{c} E_{0}^{+}\\ E_{0}^{-} \end{array}\right]=\prod_{t=1}^{3}\left\{\left[\begin{array}{cc} \left(\frac{1}{2}+\frac{\eta_{t-1}}{2\eta_{t}}\right)e^{jd_{t-1}\left(-k_{t}+k_{t-1}\right)} & \left(\frac{1}{2}-\frac{\eta_{t-1}}{2\eta_{t}}\right)e^{jd_{t-1}\left(k_{t}+k_{t-1}\right)}\\ \left(\frac{1}{2}-\frac{\eta_{t-1}}{2\eta_{t}}\right)e^{jd_{t-1}\left(-k_{t}-k_{t-1}\right)} & \left(\frac{1}{2}+\frac{\eta_{t-1}}{2\eta_{t}}\right)e^{jd_{t-1}\left(k_{t}-k_{t-1}\right)} \end{array}\right]\right\}\cdot \left[\begin{array}{c} \left(\frac{1}{2}+\frac{\eta_{3}}{2\eta_{4}}\right)e^{jd_{3}\left(-k_{4}+k_{3}\right)}\\ \left(\frac{1}{2}-\frac{\eta_{3}}{2\eta_{4}}\right)e^{jd_{3}\left(-k_{4}-k_{3}\right)} \end{array}\right]E_{4}^{+}=\left[\begin{array}{c} \alpha \\ \beta \end{array}\right]E_{4}^{+}$$

Based on Equation (4), the reflection coefficient of the slab subject to the internal hole can be readily computed by using the expression written as Equation (5):

(5) $$c_{2}=\frac{\beta }{\alpha }$$

It can be found from Equations (4) and (5) that *c*_2_ relies highly on the material properties of the slab and sizing parameters of the defect. Consequently, the internal hole could be detected and assessed by analyzing *c*_2_.

Both Equations (3) and (5) imply that the large-sized buried defect can be detected by analyzing the reflection coefficient which is correlated with the sizing parameters and electromagnetic properties of the slab. Apart from the defect size, special attention has been paid to the relative permittivity *ε*_r_ of the dielectric slab. It is noteworthy that Equations (1)–(5) hold merely under the condition that the slab is isotropic. They are inapplicable for anisotropic materials such as GFRP. However, the research by Kharkovsky et al. indicated that the apparent relative permittivity of GFRP depends on the wave-polarization angle between the microwave polarization direction of the incident wave and the fiber direction [24]. With the angle known in the testing setup, GFRP shows dielectric characteristics analogous to those of an isotropic dielectric material with the equivalent relative permittivity. This opens up the possibility of investigating the microwave response to a unidirectional GFRP with a buried defect since the anisotropic properties of GFRP can be characterized by various equivalent relative permittivity for different wave-polarization angles.

### 2.2. The Equivalent Relative Permittivity of GFRP and Simulations 

**Figure 2:**
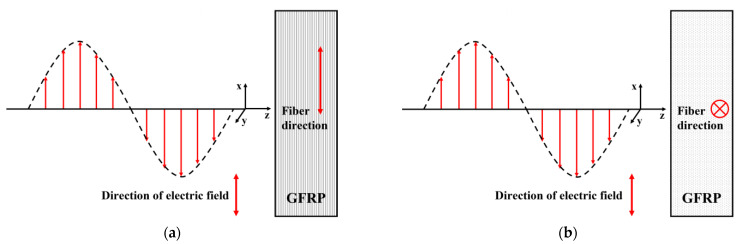
Two wave-polarization angles are involved in simulations: (**a**) the electric field direction parallel to the fiber direction; and (**b**) the electric field direction orthogonal to the fiber direction.

Two wave-polarization angles are involved in the simulations: (1) the microwave polarization direction parallel to the fiber direction of a unidirectional GFRP slab; and (2) the microwave polarization direction orthogonal to the fiber direction. The schematic illustration is presented in Figure 2.

Based on [24], for each wave-polarization-angle case, the equivalent relative dielectric can be calculated by Wiener upper limit and Hashin Shtrikman upper bound Equations (6) and (7):

(6) $$\varepsilon_{∥}=v_{f}\varepsilon_{f}+\left(1-v_{f}\right)\varepsilon_{m}$$

(7) $$\varepsilon_{⊥}=\varepsilon_{f}\left[1+\frac{3\left(1-v_{f}\right)\left(\varepsilon_{f}-\varepsilon_{m}\right)}{\left(\varepsilon_{f}-\varepsilon_{m}\right)v_{f}-3\varepsilon_{f}}\right]$$

where, $\varepsilon_{∥}$ and $\varepsilon_{⊥}$ are the equivalent relative permittivity when the GFRP is illuminated by the incident microwave with the polarization direction parallel and orthogonal to the fiber direction, respectively. $\varepsilon_{f}$ and $\varepsilon_{m}$ denote the relative permittivity of the fiber and resin, which are associated with the volume fraction *v_f_* regarding the glass fibers in the GFRP. The microwave which is reflected from the GFRP and sensed by an antenna is dependent on $\varepsilon_{∥}$ and $\varepsilon_{⊥}$. Therefore, the testing signal, aka., S_11_ acquired by a vector network analyzer (VNA) in microwave testing varies for the wave-polarization angles. It is noted that S_11_ is formulated as Equation (8):

(8) $$S_{11}=\frac{\Gamma \left(1-e^{2jkD}\right)}{1-\Gamma^{2}e^{2jkD}},\Gamma =\frac{Z_{S}-1}{Z_{S}+1},{\;Z}_{S}=\sqrt{\frac{\mu }{\varepsilon \prime }}$$

where, *D* denotes the thickness of the GFRP slab. Γ is the reflection coefficient at the heterogeneous interface. Z_s_ stands for the wave impedance in a function of magnetic permeability *μ*, *μ* = 4*π* × 10^−7^ T∙m/A and dielectric constant $\varepsilon^{\prime }$ which is either $\varepsilon_{∥}$ or $\varepsilon_{⊥}$ for each wave-polarization angle.

In reference to [27], the relative permittivity of the fiber is 6.2 whilst the relative permittivity of the resin is 3.0. The volume fraction of the fibers is normally 60% [27]. By using Equations (6) and (7), the equivalent relative permittivity of a typical unidirectional GFRP for two wave-polarization angles is $\varepsilon_{∥}$ = 4.92 and $\varepsilon_{⊥}$ = 4.77.

**Figure 3:**
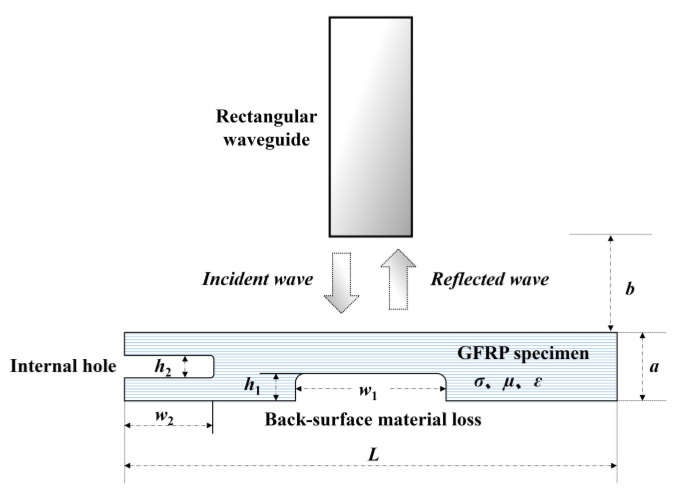
Schematic illustration of the simulation model regarding the microwave testing of a GFRP slab subject to buried defects.

With the equivalent relative permittivity computed out, further simulations were conducted in an attempt to investigate the testing signal response to the localized buried defects inspected by a Ka-band rectangular waveguide with an aperture size of 7.11 mm × 3.56 mm. In consideration of the fact that the fundamental mode of the microwave excited by waveguide is TE_10_ and only y-component of the electric field exists and its intensity is independent of y coordinate, a 2D finite element model was built up. The schematic illustration of the model is shown in Figure 3 whilst the simulation parameters are listed in Table 1.

**Figure 4:**
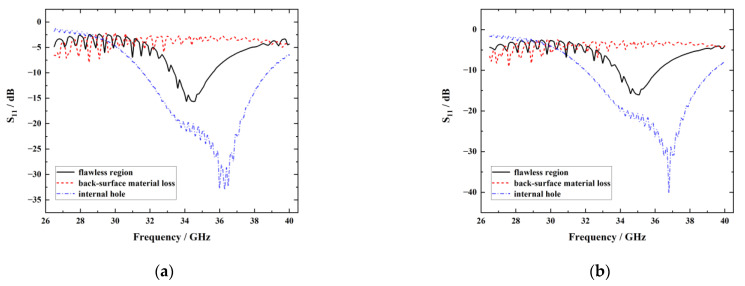
Simulated signals: (**a**) parallel; (**b**) orthogonal.

In simulations, the back surface material loss and the internal hole were individually tested by the rectangular waveguide deployed right over their centers. The number of the frequency samples was set as 136. After S_11_, each frequency sample was derived and its mean amplitude ($\bar{S_{11}}$) was subsequently calculated. The simulated testing signals, i.e., S_11_ in the function of the excitation frequency are presented in Figure 4, whilst the mean amplitudes of S_11_ are tabulated in Table 2. 

It can be seen from Figure 4 that there are obvious differences in the simulation signals at the flawless region, the back surface material loss, and the internal hole. Specifically, the S_11_ amplitude of the back surface material loss was the largest, followed by the flawless region while that of the internal hole was the smallest. It can also be seen from Table 2 that the mean amplitude of S_11_ for the back surface material loss was larger than that of the flawless region, whilst for the case of the internal hole it was the smallest. This is because compared with the flawless region, the back surface material loss reduced the loss of the incident wave as the thickness of the GFRP slab decreased, while the internal hole caused a lot of microwave scattering due to multiple interfaces. The results also imply that in the presence of buried defects, the mean amplitude of S_11_ with the microwave polarization direction parallel to the fiber direction is larger than that with the orthogonal scenario. Further analysis regarding the variation of the mean amplitude of S_11_ in the presence of buried defects reveals that it is bigger for the parallel scenario, indicating that the testing sensitivity is higher when the microwave polarization direction is parallel to the fiber direction.

## 3. Experiments for Quantitative Screening of Buried Defects

### 3.1. Experimental System Setup

**Figure 5:**
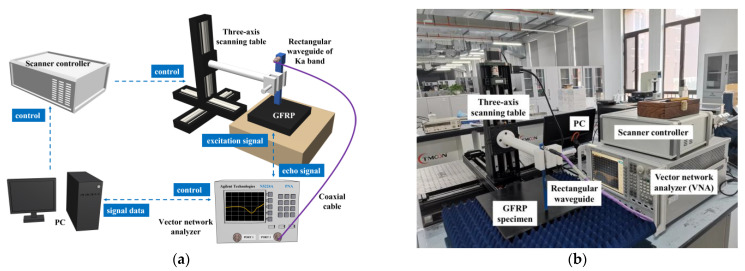
Schematic illustration of the experimental system: (**a**) the diagram; (**b**) the practical picture.

In parallel to the simulations, experiments were conducted for investigation regarding quantitative screening of buried defects in GFRP via microwave reflectometry. An experimental system of microwave testing is hereby built up. The schematic illustration of the system is portrayed in Figure 5. The system consists of a VNA (Keysight N5224A) connected with an open-ended rectangular waveguide of Ka band, three-axis scanning table, scanner controller, and PC. The Ka-band incident microwave generated by the VNA is transmitted through the coaxial cable to the waveguide, which converts the microwave from the TEM mode into TE mode. During experiments, the waveguide was deployed over the specimen with the normal wave-incident setup. It also captures the reflected wave from the specimen simultaneously. The testing signal, i.e., S_11_ was subsequently obtained from the VNA. The experimental parameters are listed in Table 3.

**Figure 6:**
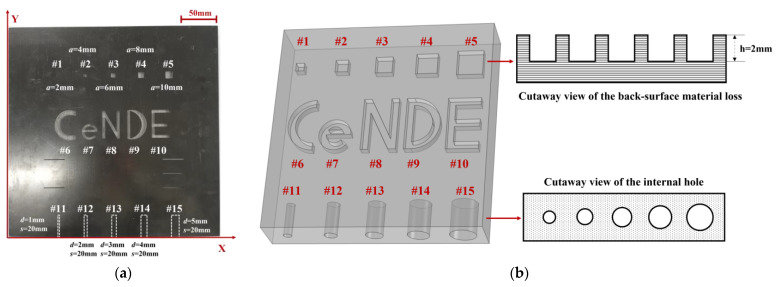
The testing specimen: (**a**) the specimen picture; (**b**) the schematic illustration of the specimen.

A planar GFRP sample containing epoxy resin and glass fiber was employed in experiments. It was fabricated with the automated fiber placement process. All the fibers in the GFRP sample were unidirectional. The sample size was 300 mm × 300 mm × 6 mm (length × width × thickness). The buried defects including the simulated back-surface material loss and internal hole were fabricated by machine work. The schematic illustration of the sample and the defects alongside their sizing parameters are exhibited in Figure 6. The back surface material loss includes two shapes: square and letter, simulating the defect with regular and irregular shapes, respectively. The internal hole was used to imitate voids in GFRP. All defect numbers and sizes are also shown in Figure 6, where *a* is the length of the square side, *h* is the depth of the material loss, *d* is the diameter of the internal hole, and *s* is the depth of the hole.

To investigate the influence of the angles between the wave-polarization direction and the fiber direction on the detection results, the rectangular waveguide was placed over the specimen with the wave-polarization angle set as either 0° (the parallel case) or 90° (the orthogonal case), which is indicated in Figure 2.

### 3.2. Defect Testing and Imaging at Different Wave-Polarization Angles

**Figure 7:**
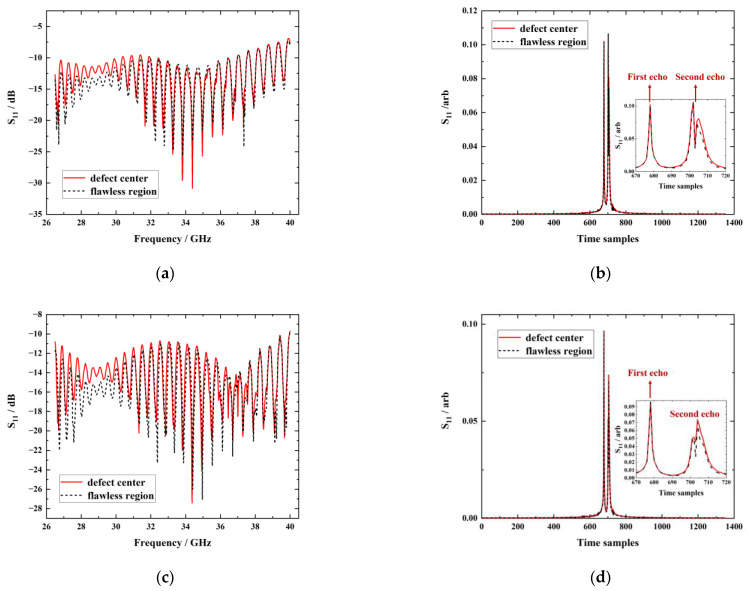
The testing signal for the flawless region and the center of the back surface material loss: (**a**) frequency–domain signals with the parallel case; (**b**) time–domain signals with the parallel case; (**c**) frequency–domain signals with the orthogonal case; (**d**) time–domain signals with the orthogonal case.

In regard to two wave-polarization angles, testing signals obtained for the flawless region and the center of the back-surface material loss are shown in Figure 7a,c. For two-angle scenarios, the amplitude of S_11_ measured at the flawless region was lower than that at the defect center in the band of 26.5 GHz~31 GHz, indicating that testing signals are responsive to the presence of buried defects. The inverse Fourier transform is exploited on frequency–domain signals in Figure 7a,c to obtain time–domain signals, as shown in Figure 7b,d. It can be observed that with both the parallel and orthogonal scenarios, the first echo of time–domain signals at the defect center and the flawless region basically overlapped whilst the second echo exhibited differences according to zoom-in plots in Figure 7b,d. The second echo of the defect center was slightly higher than that of the flawless region, indicating that the energy of the reflected wave at the defect center is greater than that of the flawless region on account of the less loss on the incident wave at the center of the back surface material loss. It is noteworthy that the amplitude of S_11_ with the wave-polarization direction parallel to the fiber direction at the flawless region and the defect center was higher than that with of orthogonal case, which is consistent with the simulation results.

**Figure 8:**
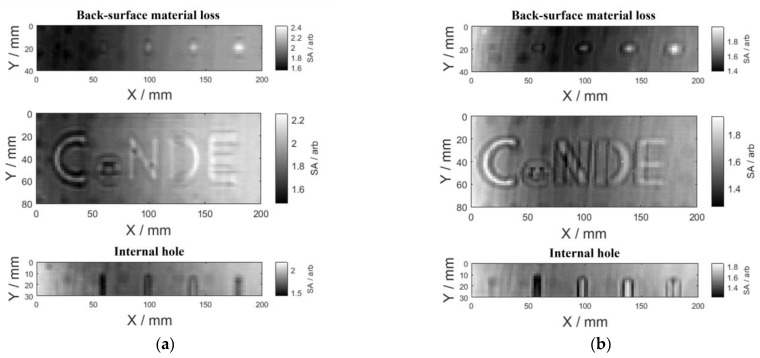
Defect images with the (**a**) parallel case and (**b**) orthogonal case.

With the 2D scanning of the specimen, after testing signals are acquired at each scanning point, the area enclosed by the time–domain signal and the time–samples axis was extracted as the signal feature, which was denoted as SA and used for defect imaging. The defect image with SA as the pixel value at every scanning point is shown in Figure 8.

It can be observed from Figure 8 that although the profile of the back-surface material loss and the internal hole can be roughly observed in the figure, the contrast between the defect region and the flawless region is low. Referring to the results presented in Figure 7, it can be found that (1) there were overlapping sections in the time–domain signals for the flawless region and the defect center; and (2) the response of the second echo to the defect was barely significant. This implies that there could be many similar magnitude-based features in the experimental signals for the defect and the flawless region. Therefore, in order to enhance the testing sensitivity and thus image contrast between the defect region and the flawless region, it is indispensable to mitigate these similar magnitude-based features within testing signals.

### 3.3. Proposition of the Direct-Wave Suppression Method

In microwave testing, the raw testing signals received by the microwave probe normally consist of (1) the reflected signal conceiving the defect information; and (2) the direct wave signal due to the coupling at the port of the rectangular waveguide along with the reflected wave from the specimen surface. Apparently, the direct wave barely conceives the information on the buried defects in the GFRP specimen. Since the magnitude of the direct wave is considerably larger than that of the reflected signal, the defect information could be masked by the redundant information of the direct wave. In other words, the testing sensitivity can be enhanced by suppressing the direct wave. 

**Figure 9:**
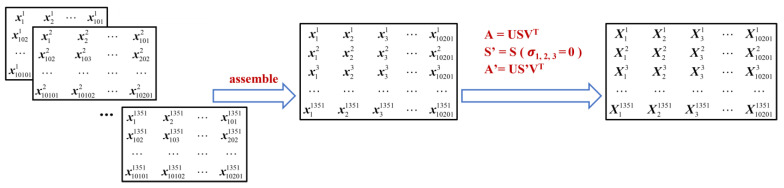
The direct wave suppression method based on SVD.

**Figure 10:**
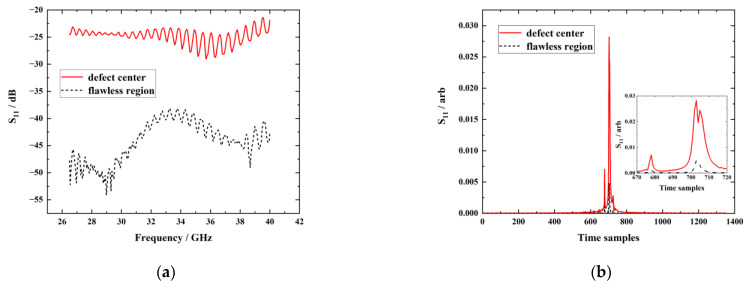

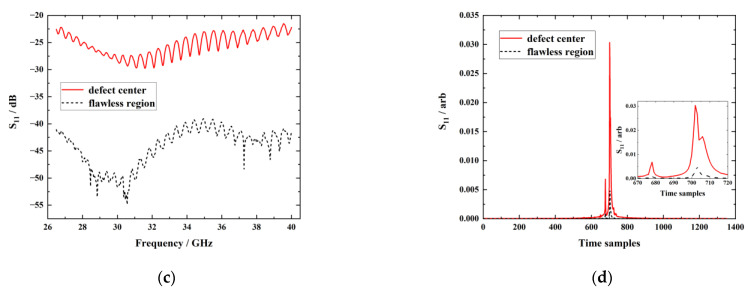
The testing signal after direct wave suppression: (**a**) frequency–domain signals with the parallel case; (**b**) time–domain signals with the parallel case; (**c**) frequency–domain signals with the orthogonal case; (**d**) time–domain signals with the orthogonal case.

In an effort to mitigate the influence of the direct wave on the testing results, particularly the defect images, a direct-wave suppression method based on single-value decomposition (SVD) is proposed. The general idea of this method is to obtain a diagonal matrix that stores single values of original reflected signals, in which the single values representing the direct-wave components are identified and subsequently set to 0. This is followed by the reflected signal being reconstructed by utilizing the diagonal matrix partially set to zero in conjunction with the inverse transformation of SVD. Specifically, the data matrix $x_{i}^{k}$ (superscript denoting frequencies and subscript standing for scanning points) is assembled into the data matrix **A** according to Figure 9 with each row in the data matrix representing reflected signals from all scanning points at the same frequency and each column representing reflected signals from all frequencies at the same scanning point. Next, single-value decomposition of the data matrix **A** is performed according to Equation (9):

(9) $$A=USV^{T}$$

where, **A** is the data matrix of the reflected signals, **U** is the left singular matrix, **V** is the right singular matrix, and **S** is the diagonal matrix with the elements on the diagonal standing for the eigenvalues of **A**, also known as singular values, arranged in descending order on the diagonal. Since according to the above analysis and relevant references the direct-wave component in the reflected signal was the strongest, the largest singular values in **S** were considered as the direct wave components and set to 0 [28]. The number of singular values set to 0 was determined by the contrast ratio of the defect image. The attempts to set the first certain number of singular values were repeated and the contrast ratio of the defect image obtained from various results of attempts was calculated. It was found that for the GFRP specimen with buried defects in this paper, when the first three largest singular values were set to 0, the contrast ratio of the defect image was highest. Hence, the direct wave components were determined to be the first three largest singular values. Ultimately, the reflected signal **A′** after suppressing the direct wave is calculated by putting the matrix **S′** partially set to zero, the matrix **U**, and the matrix **V** into Equation (9). Accordingly, testing signals corresponding to Figure 7, after the direct waves’ suppression, are shown in Figure 10.

**Figure 11:**
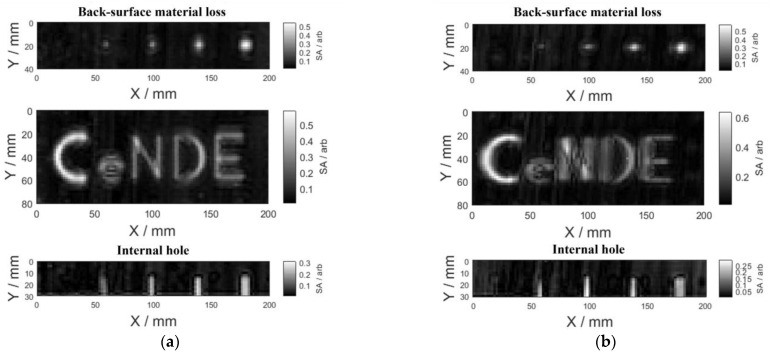
The reproduced defect images with the (**a**) parallel case and (**b**) orthogonal case.

It can be seen from the figure that after the direct wave suppression by the proposed SVD method, the frequency–domain signal and the time–domain signal at the defect center and the flawless region showed significant differences in that both the frequency–domain and time–domain signals of the flawless region were distinctly below that of the defect center. Comparing Figure 10 with Figure 7, it is noticeable that the signal difference between the defect center and the flawless region was significantly enhanced, implying the applicability of the proposed method for direct wave suppression. SA is still extracted from the reflected signal after the direct wave suppression and used for reproduction of the defect images. The reproduced defect images are shown in Figure 11.

Compared with Figure 8 and Figure 11, it can be seen from Figure 11 that the contrast between the defect region and the flawless region in the defect image was markedly promoted. However, the smallest square back-surface material loss with a side length of 2 mm and the internal hole with a diameter of 1 mm are still not presented in Figure 11, and the letter ‘e’ for back-surface material loss was relatively blurry and inaccurate. This is due to the comprehensive effect of the transverse resolution of Ka-band microwave for GFRP materials detection (the half wavelength of 40 GHz microwave in GFRP: 1.8 mm), the minimum size of the Ka-band waveguide aperture (3.56 mm), and the scanning step size (2 mm), resulting in limiting the resolution of the defect image.

The contrast ratio between the two defect images in Figure 11 was calculated to quantitatively compare the influence of the microwave polarization direction with respect to the GFRP fiber direction on the testing sensitivity, which is calculated by the ratio of the mean values of pixels in the defect region to that of the flawless region. The results are listed in Table 4. It can be found from Table 4 that the contrast ratio of defect images was higher when the wave-polarization direction is parallel to the fiber direction in testing.

### 3.4. Assessment of the Defect in-Plane Area

Based on the acquired defect images exhibited in Figure 11, a defect edge identification method is employed to obtain a quantitative evaluation of the in-plane area of buried defects. To obtain an accurate area value, it is critical to smooth the image by interpolation, and bicubic interpolation was used to increase the number of pixel points in the defect image. Subsequently, linear grayscale transformation was performed to enhance defect features in large-scale pixel images, followed by the Sobel operator being utilized to identify the defect edge in the defect image after linear grayscale transformation. The area enclosed by the defect edge curve is considered the in-plane area of buried defects. This process is abbreviated as the defect edge identification method.

**Figure 12:**
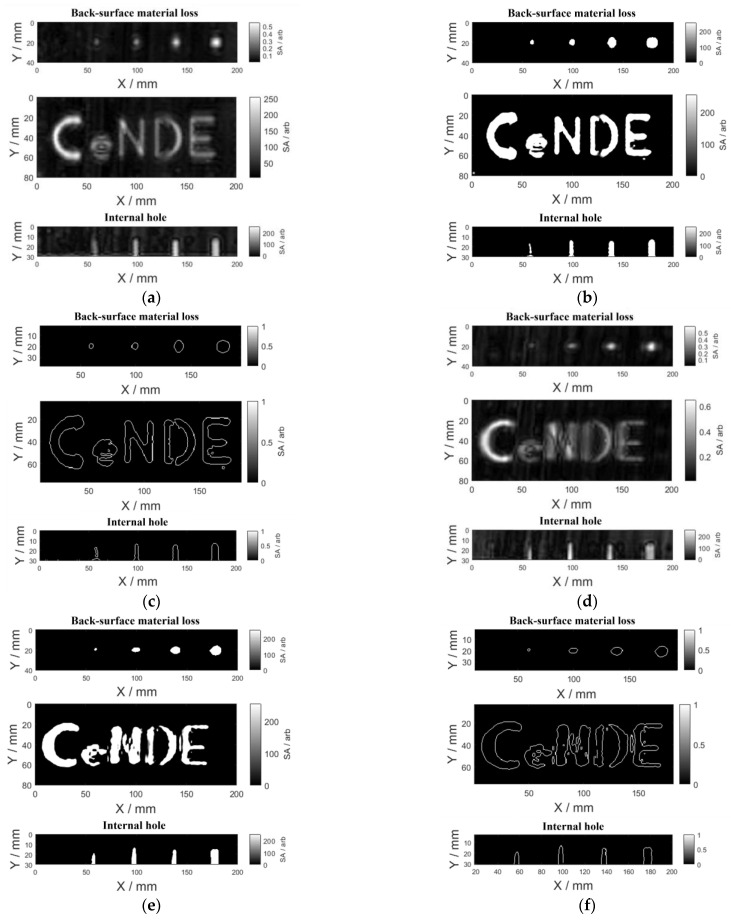
The proposed defect edge identification method: (**a**) image interpolation with the parallel case; (**b**) feature enhancement with the parallel case; (**c**) defect edge recognition with the parallel case; (**d**) image interpolation with the orthogonal case; (**e**) feature enhancement with the orthogonal case; (**f**) defect edge recognition with the orthogonal case.

The processing procedures include the following:

- Step 1: image interpolation. The number of pixel points is expanded from *W* × *H* to (*nW*) × (*nH*), so the image resolution unit is reduced from *A* to *A*/*n*^2^. The number of pixel points in Figure 11 is increased 16 times, as shown in Figure 12a,d, along with the resolution unit reducing from 2 mm × 2 mm to 0.5 mm × 0.5 mm;
- Step 2: feature enhancement. The image obtained from step 1 is converted into a grayscale image with pixel values ranging from 0 to 255, and then linear transformation on the grayscale image is performed as Equation (10):
  (10) $$J=\left(\;I-\tau \right)/\gamma$$
  where, **J** is the pixel-value matrix of the defect image after linear grayscale transformation. **I** is the pixel-value matrix of the grayscale image obtained from step 1. If the value of a certain point in **J** is less than 0, it is considered as 0, similarly, if it is greater than 255, it is considered as 255. The coefficients, i.e., *τ* and *γ* are set as 64 and 11, respectively, in Equation (10) to enhance the defect features in Figure 12a,d. The enhancement results are shown in Figure 12b,e. It can be seen that the linear grayscale transformation method can make the small values in the defect image smaller and the large values larger, thereby further suppressing the background, enhancing the pixel values at the defect region, and thus the defect features are enhanced;
- Step 3: defect edge recognition. As the Sobel operator performs weighted processing on the influence of pixel positions, it can better handle images with grayscale gradients and high noise levels. The Sobel operator is utilized to identify the defect edges in Figure 12b,e, and the identification results are shown in Figure 12c,f. The figure shows that the final defect-edge recognition effect of back surface material loss of letter shape with the microwave polarization direction parallel to the fiber direction is better and clearer than that with the orthogonal scenario.

**Figure 13:**
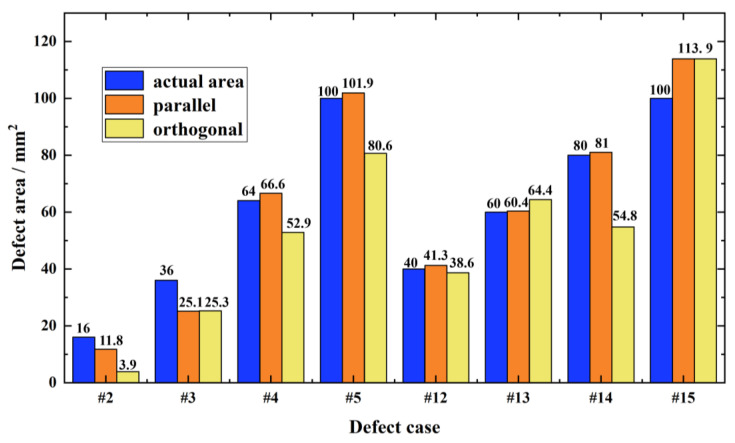
Assessment results of the in-plane areas of buried defects in GFRP.

The in-plane areas of eight detected defects with known sizes, i.e., the defects #2~#5, #12~#15, were evaluated by using the proposed defect edge identification method. The evaluation results are shown in Figure 13. 

It can be observed from Figure 13 that when the wave polarization direction is parallel to the GFRP fiber direction, the evaluated area value is closer to the actual area of the buried defect. By further calculation, the averaged relative error of evaluation results with the parallel scenario was 10.21% while the averaged relative error with the orthogonal scenario was 14.04%, which indicates that when the wave polarization direction is parallel to the GFRP fiber direction, the detection accuracy is higher. It is noteworthy that the pre-processing of the raw defect image by using the proposed direct wave suppression method is crucial and beneficial to the high evaluation accuracy regarding the in-plane area of the buried defect. 

## 4. Concluding Remarks

In this paper, the quantitative evaluation of buried defects in a unidirectional GFRP was intensively investigated via microwave reflectometry. The influence of two typical angles between the microwave polarization direction and the GFRP fiber direction, i.e., parallel and orthogonal on the testing sensitivity, was analyzed through simulations and experiments. Based on the equivalent relative permittivity corresponding to two angle scenarios calculated via theoretical analysis, a numerical simulation model was established to investigate the response of reflected signals to buried defects. In the experimental study, a microwave-reflectometry system was built for the imaging and quantitative evaluation of buried defects in GFRP. A direct wave suppression method and a defect edge identification method were proposed to improve the quality of the defect image and assess the in-plane area of buried defects, respectively. The investigation results indicated the following:

1. The simulated and experimental signals imply that regardless of the flawless region or the defect center, when the wave polarization direction is parallel to the fiber direction, the amplitude of S_11_ is greater than that with the orthogonal scenario;
2. The proposed direct wave suppression method can effectively suppress the direct-wave components in testing signals, significantly amplify the difference of signals between the defect center and the flawless region, and upgrade the quality of defect images. The minimum size of the detected defect can reach 2 mm;
3. The proposed defect edge identification method can availably evaluate the in-plane area of buried defects in GFRP. Both the contrast ratio of the defect image and the averaged relative error of assessment results regarding the defect in-plane area with two-angle scenarios imply that when the wave-polarization direction is parallel to the fiber direction, the testing sensitivity for GFRP buried defects is higher.

## Figures and Tables

**Table 1:** The modeling parameters.

Frequency Range	Stand-off Distance *b*	Specimen Size *L* × *a*	Size of the Back-Surface Wall-Thinning Defect *w*_1_ × *h*_1_	Size of the Internal Hole *w*_2_ × *h*_2_
26.5 GHz~40 GHz	1 mm	200 mm×6 mm	10 mm×3 mm	20 mm×2 mm

**Table 2:** The mean amplitude of S_11_ vs. the equivalent relative permittivity of the GFRP slab.

Equivalent Relative Permittivity	Flawless Region	Back-Surface Material Loss	Internal Hole
$\varepsilon_{∥}=4.92$	−6.23 dB	−3.89 dB	−12.41 dB
$\varepsilon_{⊥}=4.77$	−6.47 dB	−4.10 dB	−12.64 dB

**Table 3:** Parameters of the experimental setup.

Frequency Range	Frequency Samples	Scanning Interval	Stand-off Distance
26.5 GHz~40 GHz	1351	2 mm	1 mm

**Table 4:** Contrast ratio of defect images at the two wave-polarization angles.

Wave-Polarization Angle	Parallel Case	Orthogonal Case
Contrast ratio	5.7765	4.9937

## Data Availability

The data presented in this study are available on request from the corresponding author after obtaining permission of an authorized person.
